# Evaluation of the Role of Circulating Tumor Cells and Microsatellite Instability Status in Predicting Outcome of Advanced CRC Patients

**DOI:** 10.3390/jpm10040235

**Published:** 2020-11-18

**Authors:** Ippokratis Messaritakis, Maria Sfakianaki, Konstantinos Vogiatzoglou, Asimina Koulouridi, Chara Koutoulaki, Dimitrios Mavroudis, Maria Tzardi, Nikolaos Gouvas, John Tsiaoussis, John Souglakos

**Affiliations:** 1 Laboratory of Translational Oncology, Medical School, University of Crete, 70013 Heraklion, Greece; mimasf19@gmail.com (M.S.); vogiatzogloukwstas@gmail.com (K.V.); asi_minakoulouridi@yahoo.com (A.K.); xarakoutoulaki@outlook.com (C.K.); mavroudis@uoc.gr (D.M.); johnsougl@gmail.com (J.S.); 2 Department of Medical Oncology, University General Hospital of Heraklion, 71100 Heraklion, Greece; 3 Laboratory of Pathology, University General Hospital of Heraklion, 70013 Heraklion, Greece; tzardi@uoc.gr; 4 Medical School, University of Cyprus, 20537 Nicosia, Cyprus; nikos.gouvas@gmail.com; 5 Department of Anatomy, School of Medicine, University of Crete, 70013 Heraklion, Greece; tsiaoussis@uoc.gr

**Keywords:** colorectal cancer, metastatic, CTCs, CEACAM5, microsatellite instability

## Abstract

Colorectal cancer (CRC) remains one of the leading causes of cancer-related death due to its high metastatic potential. This study aimed to investigate the detection and heterogeneity of circulating tumor cells (CTCs) and the microsatellite instability (MSI) status in advanced CRC patients prior to any systemic front-line treatment. Peripheral whole blood was obtained from 198 patients. CTCs were detected using double immunofluorescence and a real time-polymerase chain reaction assay; whereas MSI status was evaluated using fragment analysis. Median age of the patients was 66 years, 63.1% were males, 65.2% had a colon/sigmoid tumor location and 90.4% had a good performance status (PS). MSI-High status was detected in 4.9% of the patients; 33.3%, 56.1% and 8.6% patients had at least one detectable CEACAM5^+^/EpCAM^+^, CEACAM5^+^/EpCAM^−^ and CEACAM5^−^/EpCAM^+^ CTC, respectively, and 9.1% of the patients had *CEACAM5*mRNA-positive CTCs. Following multivariate analysis, age, PS and MSI were confirmed as independent prognostic factors for decreased time to progression, whereas age, PS and CTC presence were confirmed as independent prognostic factors for decreased overall survival. In conclusion, our data support the use of CEACAM5 as a dynamic adverse prognostic CTC biomarker in patients with metastatic CRC and MSI-High is considered an unfavorable prognostic factor in metastatic CRC patient tumors.

## 1. Introduction

Colorectal cancer (CRC) is still one of the main causes of cancer-related death [1,2]. Its high metastatic potential is due to cancer cell dissemination through hematogenous or lymphatic vasculature.

Circulating tumor cell (CTC) presence in the blood of patients experiencing cancer has been widely described and its impact on decreased progression-free and overall survival in various cancer types has been documented with different technologies (fluorescent microscopy, molecular methods, isolation by size, CellSearch, etc.) [3,4,5,6,7,8,9,10,11,12,13]. The clinically validated CellSearch assay is based on the immunomagnetic epithelial adhesion cellular molecule (EpCAM)-positive CTC enrichment [5]. However, during their dissemination, CTCs undergo epithelial-to-mesenchymal transition and down-regulate their epithelial markers, including EpCAM [14,15]. Therefore, it is obvious that CellSearch fails to detect CTCs that do not express EpCAM.

In CRC, the carcinoembryonic antigen (CEA) is the most frequently analyzed marker, and we have previously developed a reliable and reproducible molecular method for *carcino-embryonic antigen-like cellular adhesion molecule 5* (*CEACAM5)* mRNA-positive CTC detection in CRC patients [11,13]. The presence of *CEACAM5*mRNA-positive CTCs has been associated with the worst clinical outcome, both in the metastatic and the non-metastatic setting [11,13].

An important molecular pathway in carcinogenesis is implicated in DNA repair mechanisms [16]. Errors are recognized by changes (instability) in hereditary patterns of single nucleotide sequences (microsatellites) that occur throughout the genome. Usually, the mutations refer to the hMLH1 (human mutL homolog 1) and hMSH2 (human mutS homolog 2) genes [17,18,19]. Mutations in these genes cause a phenomenon known as microsatellite instability (MSI), which occurs in most of the 10–15% of sporadic CRC cases [20,21,22]. MSI-High tumors have been shown to be associated with higher survival rates in relation to MSI-Low or MSI-Stable (MSS) tumors [23,24,25]; however, the underlying mechanism is not well understood.

The current study aimed to investigate the CTCs’ heterogeneity, based on the expression of *CEACAM5* and EpCAM markers, in advanced CRC patients prior to any systemic first-line therapy. Moreover, the study aimed to investigate the detection of CTCs expressing *CEACAM5*mRNA using the reverse transcription quantitative polymerase chain reaction (RT-qPCR) technology and the MSI status of the patients. Finally, to correlate the detection of CTCs and MSI status with known clinico-pathological characteristics and patients’ outcomes.

## 2. Materials and Methods

### 2.1. Patients’ Population

One hundred and ninety-eight patients enrolled in the study. All these patients had a newly diagnosed metastatic CRC and histologically documented disease. All 198 patients were treated at the Department of Medical Oncology, University Hospital of Heraklion (Greece). The Ethics Committee/Institutional Review Board of the University Hospital of Heraklion (Greece) has approved the current study (Number: 7302/19-8-2009). All enrolled patients have agreed their enrolment and signed their written informed consent. All procedures followed in the current study were in accordance with the ethical standards of the institutional and/or national research committee and are in agreement with the 1964 Helsinki declaration and its later amendments.

### 2.2. Blood and Tissue Samples

An amount of 15 mL in EDTA of peripheral blood was obtained at the middle-of-vein puncture. Prior collection, the first 5 mL was discarded to exclude skin epithelial cells. Isolation of peripheral blood mononuclear cells (PBMCs) was performed by Ficoll-Hypaque density (*d* = 1077 g/mL; Sigma-Aldrich, GmbH, Taufkirchen, Germany) gradient centrifugation at 1800 rpm, for 30 min. Centrifugation and cytospin preparation were described previously [26,27].

Total RNA extraction from both patients’ PBMCs and cell lines was carried out under RNAse-free conditions using Trizol (Thermo Fisher Scientific, Fremont, CA, USA) in a laminar flow hood. Following RNA isolation, it was dissolved in RNA storage buffer (Ambion, Fremont, CA, USA) and until used, it was stored at −80 °C. *β*-actin was used as house-keeping gene amplification to verify the RNA integrity, as previously described [28,29].

Formalin-fixed, paraffin-embedded (FFPE) tumor sections were evaluated by an experienced pathologist. The diagnosis was confirmed, and the tumor-enriched areas prior dissection were defined. Ten serial sections (5 µm thickness) were stained with nuclear fast red (Sigma-Aldrich, St. Louis, MO, USA). Samples with ≥80% malignant cells were scraped under the microscope. When <80% tumor cells, the piezoelectric Eppendorf microdissector (Eppendorf, Hamburg, Germany) was used for microdissection. The Epicentre^®^ Biotechnologies MasterPure™ Complete DNA and RNA Purification Kit was used for DNA extraction, according to the manufacturer’s instructions (Epicentre, Madison, WI, USA).

### 2.3. Quantitative RT-PCR (RT-qPCR) and Double Immunofluorescence

The NanoDrop (Thermo Scientific, Fremont, CA, USA) equipment was used to determine RNA concentration. The reverse transcription and qPCR conditions as well as the method’s analytical sensitivity and specificity evaluation have been described previously [11,13]. The ABI Prism 7900HT Sequence Detection System (Applied Biosystems, Waltham, MA, USA) was used to quantify gene expression (in triplicates). An external calibration curve using external standard cDNAs was used for quantification [11,13,30]. In addition, cDNA synthesis of serial dilutions of RNA isolated from the Lovo cells, corresponding to 1–10^5^ cells, was also included in each run. The number of *CEACAM5*mRNA-positive cells for all of the samples tested was expressed as Lovo cell equivalents/5 μg total RNA. The pair of primers and the probe used were as follows: CEACAM5-forward: *AATTCCATAGTCAAGAGCATCACAGT*, CEACAM5-reverse: CAGTGG CCCCAGCTGAGA, and probe: *CTGCATCTGGAACTTCTCCTGGT*. The results were analyzed using the SDS 2.3 software (Applied Biosystems, Waltham, MA, USA). Finally, as no RNA transcripts were detected in the absence of reverse transcriptase, genomic DNA contamination was excluded.

Moreover, immediately following their isolation, 5 × 10^5^ PBMCs were cyto-centrifuged on glass microscope slides, at 2000 rpm for 2 min. Cytospins were dried at room temperature and stored at −80 °C, until use. A number of 10 × 10^5^ PBMCs in total (corresponding to 2 microscope slides) from each patient were screened for CEACAM5 (Acris Antibodies, GmbH, Germany) and EpCAM (Acris Antibodies, GmbH, Germany) expression. Briefly, ice cold aceton:methanol 9:1 (*v*/*v*) was used for cell-cytospin fixation, for 20 min. All antibodies (primary and secondary) were incubated for a period of 1 h. *CEACAM5* was detected using the Zenon technology (FITC-conjugated IGg1 antibody; Molecular Probes, Invitrogen, Fremont, CA, USA); whereas EpCAM was labelled with Alexa 555 (Molecular Probes). Additionally, cytospins were double stained with the anti-CEACAM5 (mouse) or anti-EpCAM (mouse) and anti-CD45 (anti-rabbit Common Leukocyte Antigen; Santa Cruz, CA, USA) antibodies, whereas Alexa 488 (Molecular Probes) and Alexa 555 (Molecular Probes) were used for labelling, respectively. Finally, DAPI anti-fade reagent (Molecular Probes) was used for cell nuclear staining. The omission of the primary antibody was used in control experiment. The detection of CTCs was performed using a fluorescent microscope (Leica DM 2500, Heidelberg, Germany). Results are expressed as number of CTCs/10^6^ PBMCs.

### 2.4. Microsatellite Instability and Gene Mutational Analysis

Microsatellite instability (MSI) analysis was examined by PCR fragment analysis. For this assay, the five microsatellite loci (*BAT-25, BAT-26, D2S123, D5S346* and *D1S250*), according to the Bethesda panel assay recommended by the 1997 National Cancer Institute-sponsored MSI workshop, were amplified using a multiplex PCR reaction for the *BAT-25, BAT-26, D2S123, D5S346* and *D1S250* loci using the MSI Analysis System, v1.2, according to the manufacturer instructions (Promega Corp., Madison, WI, USA). A 10 μL reaction containing 40–60 ng of DNA template was prepared for each tumor and normal tissue of every patient. The PCR cycling conditions included an initial denaturation step at 95 °C (11 min) and at 96 °C (1 min), 30 cycles of denaturation at 94 °C (30 s), annealing at 60 °C (1 min) and extension at 72 °C (90 s). A final extension step at 60 °C (45 min) was added. PCR products were analyzed by capillary electrophoresis using an ABI Prism 3130 Genetic Analyzer (Thermo Fisher Scientific). In brief, deionized formamide was combined with GeneScan-500 (LIZ) size standard (Thermo Fisher Scientific) and 1.2 μL of PCR amplified product. Then, samples were denatured at 95 °C for 3 min and ice-chilled prior analysis. The GeneMapper software v4.1 (Thermo Fisher Scientific, Fremont, CA, USA) was used for data analysis. Only fragments in which both tumor and paired normal tissue DNA showed signals above background levels, were considered assessable. For interpretation purposes, MSI at ≥2 loci was defined as MSI-High, MSI at a single locus was defined as MSI-Low, whereas a locus was called stable (MSS) if no instabilities were observed in the tumor sample in comparison to the paired normal DNA of the same patient.

Sanger sequencing following PCR amplification was used for *KRAS* exon 2 mutational analysis, and an allelic discrimination assay based on real-time PCR for the *BRAF*^V600E^ mutational analysis was performed as a standard clinical practice [31,32,33]. It has to be mentioned that a retrospective *NRAS* mutation analysis was not performed due to the fact that this study was initiated prior the description of the *NRAS* predictive value in treatment efficacy [34]; a retrospective mutation analysis of this gene was not performed.

### 2.5. Study Design and Statistics

The aim of the current study was to investigate the clinical relevance of CEACAM5-positive detection and *CEACAM5*mRNA-positive CTCs on diagnosis and to correlate them with MSI and genetic mutational status. The current study has an observational nature of the study; thus, no formal sample size calculation was performed. The cut-off value of ≥1.92 Lovo cell equivalents/5 μg of RNA was used as has been previously suggested [11]. Summary tables (descriptive statistics and/or frequency tables) are provided for all timepoint variables, as appropriate. Continuous variables are demonstrated with descriptive statistics (number, median, standard deviation and range). Ninety-five percent confidence intervals (95% CI) are also mentioned. Progression-free survival (PFS) was interpreted as the time from first cycle of treatment until clinical or radiological disease relapse or any cause of death. Overall survival (OS) was defined from the date of first cycle of treatment until any cause of death or the date of last follow-up. Whenever appropriate, either Pearson’s Chi-square or Fisher’s exact test was used to compare qualitative factors. The Kruskal Wallis test was used to assess differences in continuous variables. The Kaplan-Meier analysis and log-rank test was performed to estimate PFS and OS. Cox proportional hazards regression models were used to observe associations among prognostic factors and PFS/OS. All statistical tests were two-sided. Any *p*-value < 0.05 was considered statistically significant. The SPSS statistical software, version 22.0 (SPSS Inc., Chicago, IL, USA), was used for data analysis.

## 3. Results

### 3.1. Patients’ Clinico-Pathological Characteristics

From 6/2007 to 12/2013, 198 patients with metastatic CRC were enrolled in the study. The patients’ clinico-pathological characteristics are listed in Table 1. The median age was 66 years, 125 (63.1%) patients were males, 129 (65.2%) had a colon/sigmoid tumor location, 179 (90.4%) had a good performance status (PS ECOG—Eastern Cooperative Oncology Group scale: 0–1), and 76 (39.6%) had high-grade tumors. Moreover, 148 (74.7%) patients underwent surgery and 39 (19.7%) underwent metastasectomy. *KRAS (*Kirsten rat sarcoma) exon 2 and *BRAF^V600E^* mutations were detected in 45.0% and 7.8%, respectively; whereas, MSI-High status was detected in 4.9% of the patients (Table 1 and Table S1).

### 3.2. Double Immunofluorescence for the Detection of CEACAM5^+^ and EpCAM^+^ CTCs

Following double immunofluorescence staining with anti-CEACAM5 and anti-EpCAM antibodies, 66 (33.3%), 111 (56.1%) and 17 (8.6%) patients had at least one detectable CEACAM5^+^/EpCAM^+^, CEACAM5^+^/EpCAM^−^ and CEACAM5^-^/EpCAM^+^ CTC, respectively (Table 2 and Table S1). The median number of CEACAM5^+^/EpCAM^+^, CEACAM5^+^/EpCAM^−^ and CEACAM5^−^/EpCAM^+^ CTCs were 2 (range, 1–20), 2 (range, 1–22) and 1 (range, 1–6), respectively (Table S1). There was no correlation between the patients’ characteristics and the detection of CEACAM5 and/or EpCAM positivity. Additional double immunofluorescence staining with anti-CD45 and anti-CEACAM5 or anti-EpCAM revealed no CEACAM5^+^/CD45^+^ or EpCAM^+^/CD45^+^ cells.

### 3.3. Detection of CEACAM5mRNA-Positive CTCs

According to the cut-off point (>1.91 Lovo cell equivalents/5 μg total RNA), *CEACAM5*mRNA-positivity was detected in 18 (9.1%) of the enrolled patients (Table 2 and Table S1). The median number of *CEACAM5*mRNA-positive CTCs was 3.1 (range, 1.92–71.33) (Table S1). *CEACAM5*mRNA-positivity was significantly associated with the detection of CEACAM5^+^/EpCAM^+^, CEACAM5^+^/EpCAM^−^ and CEACAM5^−^/EpCAM^+^ CTCs (*p* < 0.001; *p* < 0.001 and *p* < 0.011, respectively). In contrast, there was no correlation between the patients’ clinico-pathologic characteristics and the *CEACAM5*mRNA-positivity.

### 3.4. Patients’ Clinical Outcome According to the Genetic Mutational Profiling, MSI Status and CTC Detection

**Figure 1:**
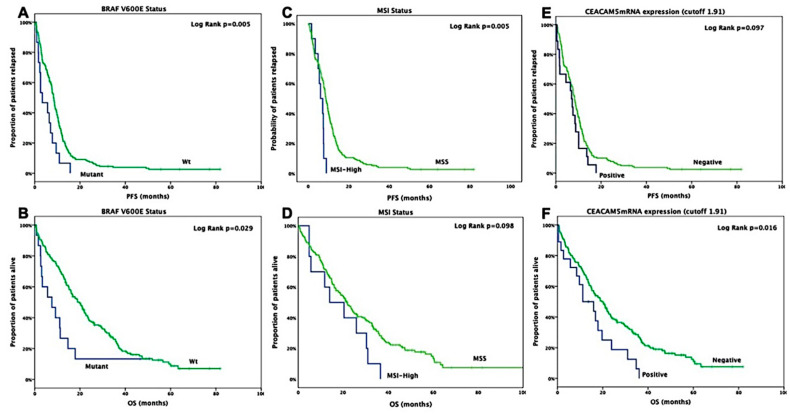
Kaplan Meier curves for progression-free and overall survival according to BRAF^*V600E*^ (**A**,**B**), microsatellite instability (**C**,**D**) and CEACAM5mRNA (**E**,**F**) status, respectively, in stage IV patients.

BRAF^*V600E*^ mutated patients presented with significantly lower PFS (3.4 months, 95% CI: 0.2–6.6 months) compared to those with BRAF^*V600E*^ wild-type tumors (8.4 months, 95% CI: 7.6–9.2 months; *p* = 0.005) (Table S1 and Figure 1A); moreover, these patients had a significantly lower OS (7.6 months, 95% CI: 0.3–14.9 months) compared to those with BRAF^*V600E*^ wild-type status tumors (19.7 months, 95% CI: 16.0–23.4 months; *p* = 0.029) (Table S1 and Figure 1B). In addition, patients with MSI-High status had a statistically significant shorter PFS (6.1 months, 95% CI: 3.5–8.7 months) compared to MSS patients (8.8 months, 95% CI: 7.9–9.7 months) (*p* = 0.005); moreover, these patients had a lower OS (14.0 months, 95% CI: 0.7–27.3 months) compared to MSS patients (21.8 months, 95% CI: 18.2–25.5 months), however, such difference was not statistically significant (*p* = 0.098) (Table S1 and Figure 1C,D). Furthermore, no significant difference was shown in patients with *CEACAM5*mRNA-positive CTCs and PFS (7.0 months, 95% CI: 5.3–8.7 months) compared to those with *CEACAM5*mRNA-negative CTCs (8.3 months, 95% CI: 7.5–9.2 months) (*p* = 0.097) (Table S1 and Figure 1E); whereas, these patients had significantly shorter OS (11.2 months, 95% CI: 2.8–19.6 months) compared to those with *CEACAM5*mRNA-negative CTCs (19.6 months, 95% CI: 15.7–23.5 months; *p* = 0.016) (Table S1 and Figure 1F). Finally, no significant difference was shown in patients with KRAS mutational status and PFS (*p* = 0.610) or OS (*p* = 0.969) (Table S1).

### 3.5. Univariate and Multivariate Analysis

Univariate analysis revealed that age, performance status (PS), BRAF^*V600E*^ and MSI status were associated with decreased PFS (Table 3). Multivariate analysis confirmed that age, PS and MSI were independent prognostic factors for decreased PFS (HR = 1.6; 95% CI: 1.1–2.2; *p* = 0.008; HR = 3.3; 95% CI: 1.8–6.0; *p* < 0.001 and HR = 2.5; 95% CI: 1.8–5.3; *p* < 0.001 (Table 3). Similarly, patients with positive *CEACAM5*mRNA CTCs had increased risk of death than those with no detectable *CEACAM5*mRNA-positive cells (HR = 1.8; 95% CI: 1.1–3.1; *p* = 0.018; Table 3). Age, PS and BRAF^*V600E*^ were also significantly associated with OS. The multivariate analysis revealed that age, PS and the presence of *CEACAM5*mRNA-positive CTCs (marginally significant) were independent prognostic factors associated with decreased overall survival (HR = 1.4; 95% CI: 1.0–1.9; *p* = 0.046; HR = 3.3; 95% CI: 2.0–5.6; *p* < 0.001 and HR = 1.6; 95% CI: 1.0–2.8; *p* = 0.056; Table 3).

## 4. Discussion

Several studies have shown that CEA has a metastatic role, which it performs through various ways [35,36,37]. CEA protects tumor cells from the effects of anoikis [38], changes the microenvironment of sinusoids, promotes the adhesion molecules’ expression and malignant cell survival, and this affords a selective advantage for tumor cell survival in circulation [36]. Furthermore, Blumenthal et al. showed that anti-CEACAM6/CEACAM5 mAb fragments targeting the *N* and A1B1 domains of these antigens lead to block migration, endothelial cells adhesion and increased survival of mice with intrapulmonary micrometastases of human colon cancer [39]. CEA has also been explored, as a target for CRC treatment and diagnosis approaches [35]. Indeed, previous studies from our group report that high *CEACAM5*mRNA levels are specifically associated with CRC progression, both in patients with operable and metastatic CRC [11,13].

In the present study, we aimed to evaluate the CEACAM5-positive CTCs expression in the blood of metastatic CRC patients, using both immunofluorescence and RT-qPCR technologies. Our results clearly revealed that positive detection of CEACAM5-mRNA was significantly correlated with the detection of CEACAM5 and/or EpCAM positivity by immunofluorescence assay, on initial diagnosis. In line with our previous results, presented data demonstrate the prognostic significance of CEACAM-positive CTCs and the BRAF^*V600E*^ mutations in metastatic CRC patients. Moreover, positive expression of *CEACAM5*mRNA was presented as an independent prognostic factor for reduced OS.

A systematic meta-analysis investigated that EpCAM expression might be associated with CRC, while its reduced expression is correlated with the progression, metastasis, and poor prognosis in CRC [40,41]. Interestingly, 56.1% of the enrolled patients had CEACAM5^+^/EpCAM^-^ detectable CTCs. These results highlight the limitation of the EpCAM-based technologies such as the Food and Drug Administration-approved CellSearch, thus failing to capture these CTC subpopulations. An explanation is that epithelial cell adhesion molecules undergoing EMT lose their epithelial and other characteristics thus acquiring a mesenchymal phenotype [42]. In addition, Kim et al. presented that any metastasizing malignant cell derived from EpCAM loss in CRC might present loss or decreased expression of EpCAM, and in these cases, CTCs cannot be detected despite being present [43]. Therefore, since epithelial markers are not always detectable [44], it might be useful to include additional biomarkers, such as the CEACAM5, thus increasing the detection sensitivity of CTCs.

MSI-High status in adjuvant CRC is associated with improved stage-specific survival; however, the prognostic role of MSI-High status in the metastatic setting remains controversial [45,46]. Several studies have shown that MSI-High metastatic CRC patients have shorter overall survival compared with MSS patients [45,47]. This phenomenon could suggest that MSI-High cancers metastasize earlier, once cancer cells evade the host immune surveillance and are about to metastasize [47]. Similarly, the current study demonstrated that patients with deficiency in the DNA mismatch repair system (MMR) have a significantly more decreased PFS than those with proficient MMR status. Moreover, our observation suggested that MSI-High is considered an unfavorable prognostic factor in metastatic CRC patient tumors.

## 5. Conclusions

In conclusion, CTCs are a promising alternative for liquid biopsy for use in precision medicine approaches [48]. Our valuable data support the use of CEACAM5 as a dynamic adverse prognostic CTC biomarker in patients with metastatic CRC; the findings should be interpreted with caution due to the limitation of the study (retrospective analysis, non-randomized cases, different treatment regimens, etc.). To our knowledge, this is the first study for the evaluation of the expression among CEACAM5, EpCAM and the MSI status of patients with metastatic CRC. Based on previous findings in a small subset of Lynch syndrome-related CRCs carrying germline EpCAM deletions [49,50], no association was found between loss of EpCAM expression and MMR system [40,43]. Accordingly, there was no significant correlation between MSI status and the expression of EpCAM or CEACAM5 in our results. Prospective studies, preferable as part of randomized trials, may further evaluate the significance of the presented biomarkers. Moreover, significant impact would be added upon evaluation of the presented biomarkers according to survival rates, both in primary tumors and metastasis.

## Figures and Tables

**Table 1:** Patients’ demographics and characteristics.

	Total (*n* = 198)	
	Frequency (*N* = 198)	%
Gender		
Male	125	63.1
Female	73	36.9
Median Age	66.3 years (20–88)	
≤70 years	121	61.1
>70 years	77	38.9
Tumor Location		
Colon/Sigmoid	129	65.2
Rectum	69	34.8
Grade		
I-II	116	60.4
III	76	39.6
Unknown	6	
Surgery		
Yes	148	74.7
No	50	25.3
Metastasectomy		
Yes	39	19.7
No	159	80.3
Performance Status (ECOG *)		
0–1	179	90.4
2	19	9.6
CEACAM5 **+/EpCAM ***+/CD45 ^#^-		
Positive	66	33.3
Negative	132	66.7
CEACAM5+/EpCAM-/CD45-		
Positive	111	56.1
Negative	87	43.9
CEACAM5-/EpCAM+/CD45-		
Positive	17	8.6
Negative	181	91.4
CEACAM5mRNA (cutoff >0.91 Lovo cell equivalents)		
Positive	18	90.9
Negative	18	9.1
*KRAS ^##^* exon 2 mutations		
Mutated	85	45
Wild type	104	55
Not Determined	9	
*BRAF*^V600E^ mutations		
Mutated	15	7.8
Wild type	177	92.2
Not Determined	6	
Microsatellite Instability (MSI)		
MSI-High	8	4.9
MSI-Stable (MSS)	155	95.1
Not Determined	35	

* ECOG: Eastern Cooperative Oncology Group; ** CEACAM5: *carcino-embryonic antigen-like cellular adhesion molecule 5*; *** EpCAM: epithelial cellular adhesion molecule; ^#^ CD45: cluster of differentiation 45; ^##^ KRAS: Kirsten rat sarcoma.

**Table 2:** Heterogeneity of circulating tumor cell (CTC) sub-populations and correlation with CEACAM5mRNA expression.

	No of Patients (*N* = 198)	%	Median	Range	*CEACAM5*mRNA
CEACAM5+/EpCAM+	66	33.3	2	1–20	*p* < 0.001
CEACAM5+/EpCAM-	111	56.1	2	1–22	*p* < 0.001
CEACAM5-/EpCAM+	17	8.6	1	1–6	*p* < 0.011
*CEACAM5*mRNA	18	9.1	3.1	1.92–71.33	

**Table 3:** Univariate and multivariate analysis for progression-free survival (PFS) and overall survival (OS).

	Univariate				Multivariate			
	PFS		OS		PFS		OS	
Feature	HR (95%CI)	*p*-Value	HR (95%CI)	*p*-Value	HR (95%CI)	*p*-Value	HR (95%CI)	*p*-Value
Age ( >70 y vs. ≤70 y)	1.4 (1.0–1.8)	0.039	1.4 (1.0–1.9)	0.043	1.6 (1.1–2.2)	0.008	1.4 (1.0–1.9)	0.046
Performance status (≥2 vs. 0–1)	4.0 (2.4–6.6)	<0.001	4.1 (2.5–6.8)	<0.001	3.3 (1.8–6.0)	<0.001	3.3 (2.0–5.6)	<0.001
KRAS exon 2	1.1 (0.8–1.4)	0.613	1.0 (0.7–1.4)	0.969	-	-	-	-
BRAF^V600E^ (mutant vs. wild type)	2.1 (1.2–3.6)	0.007	1.9 (1.1–3.3)	0.03	-	-	-	-
Microsatellite Instability (MSI) (High vs. Stable)	2.6 (1.2–5.4)	0.011	1.9 (0.9–4.0)	0.072	2.5 (1.8–5.3)	0.017	-	-
CEACAM5 **+/EpCAM ***+	1.1 (0.8–1.5)	0.394	1.2 (0.8–1.6)	0.366	-	-	-	-
CEACAM5+/EpCAM-	1.1 (0.8–1.5)	0.499	1.1 (0.8–1.4)	0.732	-	-	-	-
CEACAM5-/EpCAM+	1.2 (0.8–2.1)	0.395	1.5 (0.9–2.5)	0.15	-	-	-	-
CEACAM5mRNA (pos vs. neg)	1.5 (0.9–2.5)	0.101	1.8 (1.1–3.1)	0.018	-	-	1.6 (1.0–2.8)	0.056

** CEACAM5: carcino embryonic antigen cellular adhesion molecule 5; *** EpCAM: epithelial cellular adhesion molecule.

## Supplementary Materials

The following are available online at https://www.mdpi.com/2075-4426/10/4/235/s1, Table S1: Research Data.
