# Supplementary material for: Evaluation of the Role of Circulating Tumor Cells and Microsatellite Instability Status in Predicting Outcome of Advanced CRC Patients
Source: J Pers Med. 2020 Nov 18;10(4):235. doi: 10.3390/jpm10040235 (PMC7712177; doi:10.3390/jpm10040235)
Supplement: Supplementary file 1 [file jpm-10-00235-s001.pdf]

Patients	Gender	DOB	Age	Age_1	PS_1	Stage	CEA+/EpCAM+
1	Male	1939/7/1	71.5	>70	0-1	IV	0
2	Female	1949/7/1	60.8	<=70	0-1	IV	0
3	Female	1936/7/1	76.7	>70	0-1	IV	0
4	Female	1948/7/1	62.9	<=70	>=2	IV	0
5	Male	1941/7/1	71	>70	0-1	IV	14
6	Female	1957/7/1	55.6	<=70	0-1	IV	18
7	Female	1954/7/1	58.2	<=70	0-1	IV	20
8	Male	1952/7/19	56.1	<=70	0-1	IV	0
9	Male	1941/7/1	66.5	<=70	0-1	IV	0
10	Male	1964/1/1	46	<=70	0-1	IV	2
11	Female	1969/7/1	44.4	<=70	0-1	IV	3
12	Male	1938/7/1	75.1	>70	0-1	IV	0
13	Male	1939/7/1	68.5	<=70	0-1	IV	2
14	Female	1933/7/1	75.1	>70	0-1	IV	4
15	Female	1954/7/1	55	<=70	0-1	IV	0
16	Male	1947/7/1	61.6	<=70	0-1	IV	0
17	Male	1937/7/1	75.2	>70	0-1	IV	1
18	Male	1948/7/1	64	<=70	0-1	IV	1
19	Female	1940/7/1	70.5	>70	0-1	IV	0
20	Male	1945/7/1	66.2	<=70	0-1	IV	1
21	Female	1957/7/1	54.6	<=70	0-1	IV	0
22	Female	1932/7/1	75.3	>70	0-1	IV	2
23	Male	1930/1/1	77.9	>70	>=2	IV	0
24	Male	1932/7/1	76.6	>70	0-1	IV	0
25	Male	1945/7/1	64.5	<=70	>=2	IV	0
26	Female	1952/7/1	56.6	<=70	0-1	IV	1
27	Male	1932/7/1	80	>70	0-1	IV	0
28	Male	1940/7/1	70.7	>70	0-1	IV	3
29	Female	1943/7/1	67.7	<=70	0-1	IV	0
30	Female	1954/7/1	58.5	<=70	0-1	IV	0
31	Female	1938/7/1	75	>70	0-1	IV	1
32	Male	1967/7/1	40.6	<=70	>=2	IV	0
33	Male	1945/7/1	65.1	<=70	0-1	IV	0
34	Male	1924/7/1	88.1	>70	0-1	IV	0
35	Male	1930/1/1	79.8	>70	0-1	IV	0
36	Male	1925/7/1	84	>70	>=2	IV	0
37	Female	1930/7/1	78.9	>70	0-1	IV	0
38	Male	1976/7/1	31.9	<=70	0-1	IV	0
39	Male	1933/7/1	69.2	<=70	0-1	IV	0
40	Female	1952/7/1	55.7	<=70	0-1	IV	0
41	Female	1973/7/1	36.3	<=70	0-1	IV	0
42	Female	1949/7/1	61	<=70	0-1	IV	0
43	Male	1943/7/1	68.4	<=70	0-1	IV	0
44	Male	1940/7/1	70.2	>70	0-1	IV	0
45	Female	1961/6/20	50.1	<=70	0-1	IV	15
46	Male	1929/7/1	82.7	>70	0-1	IV	2
47	Male	1940/7/1	72.4	>70	0-1	IV	3
48	Male	1957/7/1	52.7	<=70	0-1	IV	0
49	Male	1934/7/1	73.6	>70	0-1	IV	2

50	Female	1949/7/1	63	<=70	0-1	IV	3
51	Male	1931/7/1	81.3	>70	0-1	IV	0
52	Female	1961/7/1	49	<=70	0-1	IV	0
53	Male	1935/7/1	76.1	>70	0-1	IV	9
54	Male	1991/7/1	20.4	<=70	0-1	IV	0
55	Male	1933/7/1	79.7	>70	0-1	IV	0
56	Male	1933/7/1	79.9	>70	0-1	IV	0
57	Female	1947/7/1	64.1	<=70	0-1	IV	2
58	Female	1927/7/1	81.6	>70	0-1	IV	0
59	Female	1936/12/11	71	>70	0-1	IV	6
60	Male	1925/7/1	84.8	>70	0-1	IV	0
61	Male	1940/1/1	66.3	<=70	0-1	IV	1
62	Male	1936/1/1	73.9	>70	0-1	IV	0
63	Female	1950/7/1	57.1	<=70	0-1	IV	0
64	Female	1949/7/1	61.9	<=70	0-1	IV	0
65	Male	1945/7/1	64.9	<=70	0-1	IV	0
66	Male	1943/1/1	64.5	<=70	0-1	IV	1
67	Male	1974/7/1	37.3	<=70	0-1	IV	0
68	Female	1967/7/1	43.7	<=70	0-1	IV	0
69	Male	1946/7/1	63.8	<=70	0-1	IV	0
70	Female	1938/7/1	72.2	>70	0-1	IV	0
71	Female	1962/11/10	47.7	<=70	0-1	IV	0
72	Male	1952/7/1	59	<=70	0-1	IV	0
73	Male	1934/7/1	74	>70	0-1	IV	0
74	Female	1936/7/1	71.9	>70	0-1	IV	9
75	Male	1937/7/1	75.6	>70	0-1	IV	5
76	Male	1943/7/1	64.5	<=70	0-1	IV	0
77	Female	1929/7/1	81.6	>70	0-1	IV	0
78	Female	1925/7/1	82.3	>70	>=2	IV	1
79	Male	1944/7/1	65.1	<=70	0-1	IV	0
80	Male	1955/7/1	57.6	<=70	>=2	IV	1
81	Male	1967/12/9	44.9	<=70	0-1	IV	0
82	Male	1934/1/1	74.2	>70	0-1	IV	0
83	Male	1950/7/1	57.9	<=70	0-1	IV	0
84	Female	1953/7/1	56.2	<=70	0-1	IV	1
85	Male	1960/7/1	49.2	<=70	0-1	IV	0
86	Male	1943/7/1	66.3	<=70	0-1	IV	0
87	Male	1953/7/1	59.3	<=70	0-1	IV	0
88	Male	1941/7/1	69.7	<=70	0-1	IV	0
89	Male	1977/7/1	34.2	<=70	0-1	IV	0
90	Male	1970/7/1	42.7	<=70	0-1	IV	9
91	Male	1940/7/1	69	<=70	0-1	IV	0
92	Male	1954/7/1	59.1	<=70	0-1	IV	1
93	Male	1936/7/1	74	>70	0-1	IV	0
94	Female	1949/7/1	62.1	<=70	>=2	IV	0
95	Male	1955/7/1	53.6	<=70	0-1	IV	0
96	Male	1937/7/1	73.2	>70	0-1	IV	0
97	Male	1949/7/1	64.4	<=70	0-1	IV	1
98	Male	1952/7/1	57.4	<=70	0-1	IV	0
99	Female	1968/7/1	40.5	<=70	>=2	IV	0

100	Female	1944/7/1	64.5	<=70	0-1	IV	0
101	Male	1938/7/1	73.6	>70	0-1	IV	5
102	Female	1965/7/1	42.1	<=70	0-1	IV	0
103	Female	1964/1/1	46.8	<=70	0-1	IV	0
104	Male	1944/7/1	66.3	<=70	0-1	IV	0
105	Female	1944/6/19	64.3	<=70	0-1	IV	2
106	Male	1949/7/1	59.3	<=70	0-1	IV	0
107	Female	1937/7/1	75.2	>70	0-1	IV	0
108	Male	1927/1/1	82	>70	0-1	IV	2
109	Male	1933/7/1	76.9	>70	0-1	IV	0
110	Female	1951/1/1	58	<=70	0-1	IV	0
111	Male	1949/7/1	60.3	<=70	0-1	IV	1
112	Female	1939/7/1	71.1	>70	0-1	IV	0
113	Male	1937/7/1	73.2	>70	0-1	IV	0
114	Male	1959/7/1	50.1	<=70	0-1	IV	0
115	Male	1974/7/1	37.3	<=70	0-1	IV	0
116	Male	1925/7/1	85.9	>70	0-1	IV	0
117	Male	1925/7/1	82.7	>70	0-1	IV	0
118	Female	1934/7/1	75.3	>70	0-1	IV	8
119	Male	1942/7/1	69.7	<=70	>=2	IV	0
120	Male	1942/7/1	69	<=70	0-1	IV	2
121	Female	1938/7/1	72.1	>70	0-1	IV	0
122	Male	1932/7/1	75.1	>70	0-1	IV	1
123	Male	1955/7/1	55.5	<=70	0-1	IV	0
124	Female	1945/7/1	63.7	<=70	0-1	IV	1
125	Male	1934/7/1	78.1	>70	>=2	IV	0
126	Male	1932/7/1	78.6	>70	0-1	IV	0
127	Male	1950/7/1	61.3	<=70	0-1	IV	0
128	Female	1953/7/1	55.8	<=70	0-1	IV	0
129	Male	1955/7/1	53.7	<=70	0-1	IV	0
130	Female	1938/7/1	69.6	<=70	0-1	IV	0
131	Female	1944/7/1	64.9	<=70	0-1	IV	0
132	Female	1956/4/8	51.9	<=70	0-1	IV	0
133	Female	1940/7/1	71.5	>70	0-1	IV	0
134	Male	1936/7/1	74.4	>70	0-1	IV	1
135	Female	1950/7/1	50.2	<=70	0-1	IV	0
136	Male	1952/10/26	59.6	<=70	0-1	IV	0
137	Male	1945/7/1	65.2	<=70	0-1	IV	13
138	Male	1944/7/1	68.3	<=70	0-1	IV	0
139	Female	1930/7/1	81.9	>70	0-1	IV	1
140	Male	1939/7/1	72.6	>70	0-1	IV	7
141	Male	1938/7/1	74.3	>70	0-1	IV	11
142	Male	1967/7/1	44.2	<=70	0-1	IV	19
143	Male	1944/7/1	68.7	<=70	0-1	IV	0
144	Male	1945/7/1	68.1	<=70	0-1	IV	0
145	Male	1937/7/1	73	>70	>=2	IV	0
146	Male	1942/7/1	69.4	<=70	0-1	IV	0
147	Male	1966/7/1	43.8	<=70	0-1	IV	0
148	Male	1953/7/1	56.4	<=70	0-1	IV	15
149	Male	1939/7/1	72.8	>70	0-1	IV	16

150	Male	1940/7/1	72.5	>70	0-1	IV	0
151	Male	1946/7/1	65.2	<=70	0-1	IV	0
152	Female	1923/7/1	85.7	>70	0-1	IV	0
153	Male	1956/7/1	53.7	<=70	0-1	IV	0
154	Male	1945/7/1	64.7	<=70	>=2	IV	0
155	Female	1934/10/18	77.1	>70	0-1	IV	0
156	Male	1951/7/1	61.9	<=70	0-1	IV	4
157	Female	1960/7/1	51.1	<=70	0-1	IV	0
158	Female	1967/7/1	40.5	<=70	0-1	IV	2
159	Male	1943/7/1	64	<=70	0-1	IV	0
160	Male	1954/9/17	54.8	<=70	0-1	IV	0
161	Male	1938/7/1	74.4	>70	0-1	IV	2
162	Female	1970/7/1	38.7	<=70	>=2	IV	0
163	Female	1953/7/1	58.3	<=70	0-1	IV	0
164	Male	1934/1/1	76.1	>70	0-1	IV	0
165	Female	1958/7/1	53.3	<=70	0-1	IV	14
166	Female	1935/7/1	74	>70	0-1	IV	0
167	Male	1944/1/1	63.8	<=70	0-1	IV	0
168	Male	1937/7/1	70.4	>70	0-1	IV	4
169	Male	1928/7/1	84	>70	0-1	IV	0
170	Male	1930/7/1	80.4	>70	0-1	IV	0
171	Female	1938/7/1	72	>70	0-1	IV	1
172	Male	1967/7/1	42.8	<=70	0-1	IV	1
173	Female	1935/1/1	78.5	>70	>=2	IV	1
174	Male	1952/7/1	58.6	<=70	0-1	IV	0
175	Female	1949/1/1	59.9	<=70	0-1	IV	0
176	Female	1958/7/1	55.2	<=70	0-1	IV	0
177	Female	1959/7/1	50.4	<=70	0-1	IV	0
178	Male	1930/7/27	78.6	>70	0-1	IV	0
179	Male	1953/7/1	58.6	<=70	0-1	IV	0
180	Female	1942/7/1	66.4	<=70	0-1	IV	3
181	Male	1941/7/1	71.1	>70	0-1	IV	0
182	Male	1948/7/1	62.2	<=70	0-1	IV	0
183	Female	1933/7/1	79.9	>70	0-1	IV	0
184	Female	1941/7/1	67	<=70	0-1	IV	3
185	Female	1963/7/1	47.6	<=70	0-1	IV	1
186	Female	1951/7/1	62.5	<=70	0-1	IV	0
187	Male	1941/6/2	69.1	<=70	0-1	IV	0
188	Female	1941/7/1	69	<=70	0-1	IV	2
189	Male	1930/7/1	79.9	>70	>=2	IV	0
190	Male	1949/1/28	57.8	<=70	0-1	IV	1
191	Female	1931/7/1	81.7	>70	0-1	IV	0
192	Male	1935/7/1	74.3	>70	0-1	IV	2
193	Male	1942/7/1	69	<=70	0-1	IV	0
194	Male	1949/7/1	62.6	<=70	>=2	IV	1
195	Male	1945/7/1	66.5	<=70	>=2	IV	7
196	Male	1932/7/1	75.2	>70	>=2	IV	0
197	Male	1943/7/1	67	<=70	0-1	IV	1
198	Male	1931/1/1	79.2	>70	0-1	IV	2

CEA+/EpCAM-	CEA-/EpCAM+	KRAS	BRAF	MSI Status	Grade
1	0	Mutant	WT		III
0	0	Mutant	WT	MSS	II
0	0	WT	WT		III
0	0	WT	Mutant		
2	1				II
8	1	Mutant	WT		III
14	4	Mutant	WT		II
0	0	Mutant	WT		II
1	0	Mutant	WT		III
1	6	Mutant	WT	MSS	II
4	0	WT	Mutant	MSS	III
0	0	WT	WT		II
1	1	Mutant	WT		II
6	0	WT	Mutant		II
2	0	WT	WT		III
0	0	Mutant	WT	MSS	II
1	0	WT	WT	MSS	II
5	1	WT	WT		II
0	0	Mutant	WT	MSS	II
3	0	WT	WT		II
4	0	Mutant	WT		II
1	0	WT	Mutant		III
2	0	WT	WT		II
0	0	Mutant	WT		II
4	0	WT	Mutant	MSS	III
1	0	Mutant	WT	MSS	II
0	0	WT	WT	MSS	II
12	0	Mutant	WT	MSS	III
0	0	Mutant	WT		III
2	0	Mutant	WT		II
3	0	WT	WT		III
0	0	Mutant	WT		III
1	0	WT	WT	MSS	III
0	0	WT	WT	MSS	III
1	0	WT	WT		II
0	0	WT	WT	MSS	III
2	0	Mutant	WT	MSS	III
1	0	Mutant	WT	MSI-High	III
2	0	WT	WT	MSS	III
4	0	Mutant	WT	MSS	II
0	0	WT	WT	MSS	II
1	0	Mutant	WT	MSS	II
2	0	WT	WT	MSS	II
0	0	WT	WT	MSS	
6	0	Mutant	WT		II
1	0	Mutant	WT	MSS	II
1	0	WT	WT		II
0	0	WT	WT		II
4	1	WT	WT	MSS	II

0	0	Mutant	WT		III
3	0	Mutant	WT		II
0	0	WT	WT		III
0	0	WT	WT		II
2	0	WT	WT	MSS	III
0	0	Mutant	WT	MSS	II
0	0	WT	WT	MSS	III
2	0	WT	WT		II
0	0	WT	WT		II
3	0	WT	WT	MSS	II
8	0	Mutant	WT		II
4	0	WT	WT	MSS	II
3	0	Mutant	WT	MSS	II
0	0		WT		II
0	0	Mutant	WT		III
0	0	WT	WT	MSS	II
4	1	WT	WT		II
1	0	WT	WT		III
1	0	Mutant	WT		II
1	0	Mutant	WT	MSS	II
0	0	WT	WT	MSS	II
0	0	Mutant	WT	MSS	III
0	0	Mutant	Mutant	MSS	III
0	0	WT	WT	MSS	II
4	0	WT	WT		III
0	0	WT	WT	MSS	III
2	0	WT	WT		II
0	0	WT	WT		II
3	0		WT		III
0	0	Mutant	Mutant	MSS	III
3	0	WT	WT		II
4	0	Mutant	WT		III
0	0	WT	Mutant		II
0	0	WT	WT		III
1	0	Mutant	WT		III
0	0	Mutant	WT		II
0	0	Mutant	Mutant		III
0	0	WT		MSS	III
2	0	WT	WT	MSS	II
0	0	Mutant	WT		
22	4	WT			II
0	0	Mutant	WT		II
1	0	WT			III
1	0	Mutant	WT		II
4	0	WT	Mutant		II
2	0	Mutant	WT		III
0	0	Mutant	WT	MSS	III
2	0	WT	WT	MSS	III
0	0	WT			III
0	0	WT	WT	MSS	III

0	0	WT	WT	MSS	II
3	0	Mutant	WT		II
0	0	WT	WT		III
2	0	WT	WT		III
2	0	Mutant	WT	MSS	II
0	0	WT	WT		II
1	0	WT	WT	MSS	III
1	0	Mutant	WT	MSS	II
0	1	WT	WT		II
1	0	WT	WT		III
0	0	Mutant	WT	MSS	I
3	0	Mutant	WT		II
0	0	WT	WT	MSS	II
0	0	WT	WT	MSS	II
2	0	WT	WT	MSS	II
0	0	Mutant	WT	MSS	II
0	0	Mutant	WT		II
0	0	Mutant	WT	MSS	II
4	0	Mutant	WT	MSS	II
0	0	WT	Mutant		II
4	1	Mutant	WT	MSS	
0	0	WT	WT	MSS	II
2	1	WT	WT		III
0	0	Mutant	WT	MSS	III
0	1	WT	WT	MSS	III
2	0	WT	WT	MSS	III
2	0	Mutant	WT		II
2	0	Mutant	WT		III
0	0	Mutant	WT		II
1	0	WT	WT		III
1	0	WT	WT	MSS	III
0	0	Mutant	WT	MSS	II
3	0	Mutant	WT	MSS	II
0	0	WT	WT		II
2	0	WT	WT		III
2	0	Mutant	WT		II
0	0	WT	Mutant	MSS	III
7	0	WT	WT		III
8	0	Mutant	WT	MSS	II
1	0	WT	Mutant	MSS	II
6	0	WT			II
9	0	Mutant	WT		III
12	0	WT	WT	MSS	III
0	0	WT	WT		II
1	3	WT	WT	MSS	III
0	0	Mutant	WT		II
0	0	WT	WT	MSS	II
0	0	Mutant	WT		III
4	3	Mutant	WT	MSS	II
11	0	Mutant	WT	MSS	

0	0	WT	WT	MSS	II
0	0	Mutant	WT	MSI-High	III
0	0		WT		II
0	0	WT	WT	MSS	III
0	0	WT	WT	MSS	I
1	0	WT	WT		III
1	0	Mutant	WT		III
0	0	WT	WT	MSS	II
1	0	WT	WT		II
0	0	Mutant	WT	MSS	III
0	0	WT	WT	MSS	III
5	0	Mutant	WT	MSS	III
0	0	WT	WT		II
0	0	WT	WT	MSI-High	II
0	0	WT	WT	MSS	II
5	0	WT	WT	MSS	III
0	0		WT		II
0	0	WT	WT	MSS	II
2	0		WT		III
0	0	WT	WT	MSS	II
0	0	Mutant	WT	MSS	II
3	0	WT	WT	MSS	III
2	0	Mutant	WT		I
1	0	WT	WT	MSS	II
0	0	Mutant	WT	MSS	II
0	0	Mutant	WT		II
2	0	Mutant	WT	MSS	III
4	0	WT	Mutant		III
0	0	WT	WT		II
1	0	Mutant	WT		II
7	2	WT	WT		III
3	0	Mutant	WT	MSS	II
2	0	Mutant	WT	MSS	II
0	0	WT	WT		I
5	0	Mutant	WT		II
9	0	WT	Mutant	MSS	III
3	0	Mutant	WT	MSS	III
0	0	Mutant	WT	MSS	II
6	0	WT	WT		II
2	0	WT	WT	MSI-High	II
0	0		WT		III
1	0		WT		II
4	0	Mutant	WT		
0	0	Mutant	WT		II
2	0	Mutant	WT	MSS	III
11	2	WT	WT	MSS	II
0	0		WT		II
3	0	WT	WT	MSS	II
3	0	Mutant	WT	MSS	II

Metastasectomy	Primary	Surgery	Baseline Date
no	TRANSVERSE/DESCENDING	No	2010/12/22
yes	ASCENDING	Yes	2010/4/16
no	COLON	Yes	2013/3/26
no	RECTUM	No	2011/5/26
no	RECTUM	No	2012/7/19
no	RECTUM	No	2013/2/18
no	SIGMOID	Yes	2012/9/10
no	RECTUM	No	2008/9/9
no	RECTUM	No	2007/12/20
no	RECTUM	Yes	2009/12/21
no	COLON	Yes	2013/12/12
no	RECTUM	Yes	2013/8/5
no	RECTUM	Yes	2007/12/21
no	RECTUM	No	2008/8/5
yes	RECTUM	Yes	2009/6/23
no	RECTUM	Yes	2009/2/20
no	SIGMOID	Yes	2012/9/26
no	SIGMOID	No	2012/7/9
no	RECTUM	Yes	2010/12/27
no	RECTUM	Yes	2011/9/21
no	CECUM	No	2012/2/14
no	COLON	Yes	2007/10/22
no	RECTUM-SIGMOID	Yes	2007/11/26
no	RECTUM	Yes	2009/2/11
no	RECTUM	Yes	2010/1/11
yes	COLON	Yes	2009/2/11
no	RECTUM	No	2012/6/19
no	RECTUM	Yes	2011/3/30
no	SIGMOID	No	2011/3/22
yes	RECTUM	No	2012/12/27
no	COLON	Yes	2013/7/15
no	RECTUM	No	2008/2/20
no	COLON	No	2010/7/28
no	SIGMOID	Yes	2012/8/1
no	COLON	Yes	2009/10/26
no	ASCENDING	Yes	2009/6/25
no	SIGMOID	Yes	2009/6/11
no	Transverse	Yes	2008/5/28
no	Colon	Yes	2002/8/26
yes	SIGMOID	Yes	2008/3/12
yes	RECTUM	No	2009/10/20
no	SIGMOID	Yes	2010/7/12
no	RECTUM	Yes	2011/11/7
yes	SIGMOID	No	2010/9/24
no	CECUM	Yes	2011/8/12
no	SIGMOID/RECTUM	Yes	2012/3/9
no	SIGMOID	No	2012/11/14
yes	RECTUM	Yes	2010/3/9
no	SIGMOID	Yes	2008/1/30

no	SIGMOID	Yes	2012/6/25
no	SIGMOID	Yes	2012/10/9
yes	SIGMOID	Yes	2010/6/16
no	RECTUM/SIGMOID	No	2011/8/11
no	Descending	Yes	2011/11/14
no	COLON	Yes	2013/3/8
no	SIGMOID	Yes	2013/6/12
no	RECTUM	No	2011/8/11
no	RECTUM	Yes	2009/1/27
no	RECTUM	No	2007/12/20
no	RECTUM	Yes	2010/4/26
yes	RECTO-SIGMOID	Yes	2006/4/27
no	RECTUM	Yes	2009/11/10
no	SIGMOID	No	2007/8/14
no	ASCENDING	No	2011/6/2
no	SIGMOID	Yes	2010/5/25
yes	ASCENDING/TRANSVERSE	Yes	2007/6/18
no	Descending	Yes	2011/10/24
no	RECTUM/SIGMOID	Yes	2011/2/24
yes	RECTUM	Yes	2010/5/4
yes	RECTUM/SIGMOID	Yes	2010/9/16
no	COLON	Yes	2010/8/2
no	DESCENDING/SIGMOID	Yes	2011/6/20
no	RECTUM	Yes	2008/6/26
no	ASCENDING	Yes	2008/6/6
no	RECTUM	Yes	2013/2/22
no	SIGMOID	No	2007/12/27
no	RECTUM	Yes	2011/2/2
no	RECTUM	No	2007/10/30
no	SIGMOID	Yes	2009/8/19
no	RECTUM	Yes	2013/1/30
yes	SIGMOID	Yes	2012/11/5
no	CECUM	Yes	2008/3/7
no	SIGMOID	Yes	2008/5/30
no	RECTUM	Yes	2009/9/29
no	CECUM	No	2009/9/2
no	SIGMOID	Yes	2009/10/2
no	RECTUM	Yes	2012/10/23
no	RECTUM	Yes	2011/2/28
no	Descending	Yes	2011/9/18
no	RECTUM	Yes	2013/3/22
no	RECTUM	No	2009/7/7
no	RECTUM	Yes	2013/8/5
yes	SIGMOID	No	2010/7/5
no	COLON	No	2011/8/5
no	CECUM	Yes	2009/2/5
no	SIGMOID/RECTUM	Yes	2010/8/30
no	RECTUM	Yes	2013/11/25
yes	COLON	Yes	2009/11/25
no	ASCENDING	Yes	2008/12/19

yes	RECTUM	Yes	2008/12/19
no	SIGMOID	Yes	2012/2/9
no	RECTUM	Yes	2007/7/20
yes	SIGMOID	Yes	2010/10/25
no	ASCENDING	Yes	2010/10/4
yes	DESCENDING/TRANSVERSE	Yes	2008/9/29
no	COLON	Yes	2008/10/10
no	COLON	Yes	2012/9/24
no	RECTUM	No	2008/12/29
no	RECTUM	Yes	2010/5/10
yes	SIGMOID	Yes	2008/12/19
no	SIGMOID	Yes	2009/10/26
no	SIGMOID	Yes	2010/8/16
no	Transverse	Yes	2010/9/27
no	RECTUM	Yes	2009/8/10
yes	RECTUM	Yes	2011/10/12
no	RECTUM	No	2011/5/9
no	RECTUM	No	2008/3/7
no	CECUM	Yes	2009/10/16
no	RECTUM	Yes	2012/3/20
no	RECTUM	No	2011/7/6
yes	RECTUM	Yes	2010/8/10
no	SIGMOID	Yes	2007/8/14
no	ASCENDING	Yes	2010/12/15
no	COLON	Yes	2009/3/23
no	SIGMOID	Yes	2012/8/8
no	RECTUM/SIGMOID	Yes	2011/2/20
no	RECTUM	No	2011/10/19
yes	SIGMOID	Yes	2009/4/6
yes	RECTUM	Yes	2009/3/17
no	SIGMOID	Yes	2008/1/23
no	RECTUM/SIGMOID	Yes	2009/5/14
no	SIGMOID	No	2008/3/4
no	SIGMOID	Yes	2012/1/9
no	ASCENDING	No	2010/11/26
no	RECTUM	No	2000/9/18
no	SIGMOID	Yes	2012/5/23
no	ASCENDING	Yes	2010/8/30
yes	SIGMOID	Yes	2012/10/8
no	RECTUM	No	2012/6/5
no	SIGMOID/RECTUM	Yes	2012/1/31
no	RECTUM	Yes	2012/10/24
no	ASCENDING	Yes	2011/8/26
yes	SIGMOID/RECTUM	Yes	2013/2/26
no	SIGMOID	Yes	2013/7/22
no	SIGMOID	No	2010/6/13
yes	SIGMOID	Yes	2011/12/5
no	RECTUM	Yes	2010/4/28
no	Descending	No	2009/11/30
no	COLON	No	2012/5/2

yes	Sigmoid	Yes	2012/12/18
no	CECUM	Yes	2011/9/2
no	COLON	Yes	2009/3/23
no	Descending	Yes	2010/3/23
no	SIGMOID	Yes	2010/3/30
no	CECUM	Yes	2011/11/10
yes	COLON	No	2013/5/20
yes	SIGMOID	Yes	2011/8/12
no	SIGMOID	Yes	2008/1/7
no	RECTUM	Yes	2007/7/11
no	SIGMOID	Yes	2009/7/13
no	Descending	Yes	2012/11/26
no	ASCENDING	Yes	2009/3/16
no	SIGMOID	No	2011/10/6
yes	COLON	Yes	2010/2/22
no	RECTUM	No	2011/10/20
no	SIGMOID	No	2009/7/10
yes	SIGMOID	Yes	2007/10/30
no	RECTO-SIGMOID	Yes	2007/11/26
no	SIGMOID	Yes	2012/7/3
no	SIGMOID	Yes	2010/11/18
yes	RECTUM/SIGMOID	Yes	2010/6/21
no	APPENDIX (BLENNΩΔΕΣ ΑΔENOCA)	Yes	2010/4/13
no	RECTUM	No	2013/7/1
yes	RECTUM	Yes	2011/1/27
no	SIGMOID	Yes	2008/12/5
yes	ASCENDING	Yes	2013/9/25
no	COLON	Yes	2009/12/7
no	SIGMOID	Yes	2009/2/27
no	SIGMOID	Yes	2012/2/2
no	Transverse	Yes	2008/11/6
no	RECTUM	No	2012/7/23
yes	RECTUM	Yes	2010/9/22
no	RECTUM	No	2013/6/12
no	RECTUM	No	2008/6/19
no	RECTUM	Yes	2011/2/1
yes	RECTUM	Yes	2013/12/27
no	CECUM	Yes	2010/7/9
no	SIGMOID	Yes	2010/6/23
no	RECTUM	Yes	2010/5/25
no	ASCENDING	No	2006/11/29
no	RECTUM	Yes	2013/3/26
yes	UNKNOWN	No	2009/11/2
no	TRANSVERSE	Yes	2011/7/18
no	SIGMOID	Yes	2012/2/7
yes	SIGMOID	Yes	2012/1/18
no	SIGMOID	Yes	2007/9/19
no	RECTUM	Yes	2010/6/25
no	DESCENDING	Yes	2010/3/1

CEACAM5mRNA baseline	qPCR Cutoff_1.91	CEACAM5 result	CEACAM5 Immunofluorescence
0	<1.9	Neg	0
0	<1.9	Neg	3
0	<1.9	Neg	
0	<1.9	Neg	0
0	<1.9	Neg	0
0	<1.9	Neg	
0	<1.9	Neg	0
0	<1.9	Neg	0
0	<1.9	Neg	0
0.93	<1.9	Pos	0
0	<1.9	Neg	
0	<1.9	Neg	
1.92	>=1.92	Pos	0
11.48	>=1.92	Pos	
0	<1.9	Neg	0
0	<1.9	Neg	0
0.38	<1.9	Neg	0
0	<1.9	Neg	0
0	<1.9	Neg	0
0.85	<1.9	Pos	0
6.22	>=1.92	Pos	
6.87	>=1.92	Pos	
0	<1.9	Neg	0
0	<1.9	Neg	0
0	<1.9	Neg	0
0.34	<1.9	Neg	0
0	<1.9	Neg	0
0.41	<1.9	Neg	0
0	<1.9	Neg	0
0	<1.9	Neg	
1.93	>=1.92	Pos	
0	<1.9	Neg	0
1.2	<1.9	Pos	
0	<1.9	Neg	0
0	<1.9	Neg	0
0	<1.9	Neg	0
0	<1.9	Neg	0
0	<1.9	Neg	
0	<1.9	Neg	
0	<1.9	Neg	
0	<1.9	Neg	0
0	<1.9	Neg	0
0.79	<1.9	Pos	0
0	<1.9	Neg	0
0.16	<1.9	Neg	0
0	<1.9	Neg	0
0	<1.9	Neg	
0	<1.9	Neg	0
2.67	>=1.92	Pos	0

0	<1.9	Neg	0
0	<1.9	Neg	0
0	<1.9	Neg	0
0	<1.9	Neg	0
0.98	<1.9	Pos	1
0	<1.9	Neg	
0	<1.9	Neg	
0	<1.9	Neg	0
0	<1.9	Neg	0
2.1	>=1.92	Pos	0
0	<1.9	Neg	0
0.93	<1.9	Pos	1
0	<1.9	Neg	0
0	<1.9	Neg	0
0	<1.9	Neg	0
0	<1.9	Neg	0
0.83	<1.9	Pos	1
71.33	>=1.92	Pos	40
0	<1.9	Neg	0
0	<1.9	Neg	0
0	<1.9	Neg	0
0	<1.9	Neg	
0	<1.9	Neg	0
0	<1.9	Neg	
2.02	>=1.92	Pos	0
0.69	<1.9	Neg	
1.09	<1.9	Pos	0
0	<1.9	Neg	0
0	<1.9	Neg	0
0	<1.9	Neg	0
2.39	>=1.92	Pos	
0	<1.9	Neg	
0	<1.9	Neg	0
0	<1.9	Neg	0
0	<1.9	Neg	0
0	<1.9	Neg	0
0	<1.9	Neg	0
0	<1.9	Neg	
1.14	<1.9	Pos	1
0	<1.9	Neg	0
0	<1.9	Neg	0
0	<1.9	Neg	0
0.7	<1.9	Neg	
0.65	<1.9	Neg	
1.8	<1.9	Pos	2
0.86	<1.9	Pos	1
0	<1.9	Neg	
0.71	<1.9	Pos	
0	<1.9	Neg	0
0	<1.9	Neg	0

8.61	>=1.92	Pos	9
1	<1.9	Pos	1
0	<1.9	Neg	0
0	<1.9	Neg	0
0	<1.9	Neg	0
0	<1.9	Neg	0
0	<1.9	Neg	0
0.76	<1.9	Pos	0
0	<1.9	Neg	0
0.72	<1.9	Pos	
0	<1.9	Neg	0
0.71	<1.9	Pos	0
0	<1.9	Neg	
0	<1.9	Neg	0
0	<1.9	Neg	0
0	<1.9	Neg	0
0	<1.9	Neg	0
0	<1.9	Neg	0
0	<1.9	Neg	0
0	<1.9	Neg	0
5.2	>=1.92	Pos	2
0	<1.9	Neg	0
1.96	>=1.92	Pos	0
0	<1.9	Neg	
0	<1.9	Neg	0
0.58	<1.9	Neg	0
0.52	<1.9	Neg	1
0	<1.9	Neg	0
0	<1.9	Neg	0
0	<1.9	Neg	0
0.61	<1.9	Neg	0
0	<1.9	Neg	0
0	<1.9	Neg	0
0	<1.9	Neg	0
0.25	<1.9	Neg	0
0	<1.9	Neg	0
0	<1.9	Neg	0
0	<1.9	Neg	0
0	<1.9	Neg	
8.74	>=1.92	Pos	
0	<1.9	Neg	0
0.53	<1.9	Neg	
0.39	<1.9	Neg	0
0	<1.9	Neg	
0	<1.9	Neg	
0	<1.9	Neg	0
0	<1.9	Neg	0
0	<1.9	Neg	
0	<1.9	Neg	0
0	<1.9	Neg	

0	<1.9	Neg	0
0	<1.9	Neg	0
0	<1.9	Neg	0
0	<1.9	Neg	0
0	<1.9	Neg	
0	<1.9	Neg	0
0	<1.9	Neg	
0	<1.9	Neg	
0	<1.9	Neg	0
0	<1.9	Neg	0
0	<1.9	Neg	0
3.52	>=1.92	Pos	
0	<1.9	Neg	0
0	<1.9	Neg	0
0	<1.9	Neg	0
0.33	<1.9	Neg	0
0	<1.9	Neg	0
0	<1.9	Neg	0
0.8	<1.9	Pos	0
0	<1.9	Neg	0
0	<1.9	Neg	0
0	<1.9	Neg	0
0.85	<1.9	Pos	0
1.06	<1.9	Pos	
0	<1.9	Neg	0
0	<1.9	Neg	0
0.68	<1.9	Neg	
0	<1.9	Neg	0
0	<1.9	Neg	0
0.76	<1.9	Pos	
0.78	<1.9	Pos	2
0	<1.9	Neg	0
1.8	<1.9	Pos	2
0	<1.9	Neg	
0	<1.9	Neg	0
1.23	<1.9	Pos	1
2.1	>=1.92	Pos	
0	<1.9	Neg	0
2	>=1.92	Pos	
0	<1.9	Neg	
0.92	<1.9	Pos	0
0.54	<1.9	Neg	
0	<1.9	Neg	0
0	<1.9	Neg	0
0	<1.9	Neg	0
8.19	>=1.92	Pos	
0	<1.9	Neg	0
1	<1.9	Pos	
0	<1.9	Neg	0

Response	Relapse	Relapse_D	PFS	survival_date	Status	OS
PD	Yes	2011/3/31	3.3	2011/10/1	Dead	9.3
Uknown	Yes	2012/3/5	22.6	2015/4/28	Alive	60.4
PD	Yes	2013/9/10	5.5	2014/8/22	Dead	16.9
PD	Yes	2011/9/7	3.4	2011/9/7	Dead	3.4
PR	Yes	2013/9/23	14.2	2014/3/7	Dead	19.6
SD	Yes	2013/8/12	5.7	2014/8/15	Dead	17.8
PD	Yes	2013/2/25	5.5	2013/9/4	Dead	11.8
SD	Yes	2009/9/14	12.2	2011/10/27	Dead	37.6
SD	Yes	2008/12/30	12.4	2011/12/1	Dead	47.4
PR	Yes	2011/3/8	14.5	2012/3/1	Dead	26.3
PD	Yes	2014/2/28	2.6	2014/9/17	Dead	9.2
SD	Yes	2014/6/23	10.6	2014/12/31	Alive	16.9
SD	Yes	2008/8/8	7.6	2010/7/22	Dead	31
PD	Yes	2008/9/2	0.9	2008/9/22	Dead	1.6
PR	Yes	2010/7/30	13.2	2014/5/30	Dead	59.2
PR	Yes	2010/7/12	16.7	2013/11/10	Dead	56.6
PR	Yes	2013/2/24	5	2013/2/24	Dead	5
PD	Yes	2012/9/5	1.9	2012/9/6	Dead	1.9
PR	Yes	2015/2/2	49.2	2015/2/2	Alive	49.2
PR	Yes	2013/12/19	26.9	2015/5/4	Alive	43.4
PD	Yes	2012/2/29	0.5	2012/2/29	Dead	0.5
PD	Yes	2007/12/14	1.7	2008/4/13	Dead	5.7
PD	Yes	2008/2/21	2.9	2009/6/24	Dead	18.9
PD	Yes	2009/3/10	0.9	2009/4/15	Dead	2.1
PD	Yes	2010/2/1	0.7	2010/2/1	Dead	0.7
SD	Yes	2010/2/25	12.5	2013/6/11	Dead	51.9
PR	Yes	2013/6/3	11.5	2015/3/19	Alive	33
PD	Yes	2011/7/14	3.5	2012/4/30	Dead	13
PD	Yes	2011/6/7	2.5	2011/6/21	Dead	3
PR	Yes	2014/4/7	15.3	2015/3/4	Alive	26.2
SD	Yes	2014/4/8	8.8	2015/1/10	Dead	17.9
PD	Yes	2008/4/8	1.6	2008/5/1	Dead	2.3
SD	Yes	2011/9/15	13.6	2012/9/19	Dead	25.8
PD	Yes	2012/10/8	2.2	2013/1/1	Dead	5
SD	Yes	2010/11/10	12.5	2012/3/9	Dead	28.4
PD	Yes	2009/9/1	2.2	2009/9/1	Dead	2.2
SD	Yes	2010/4/28	10.5	2010/4/28	Dead	10.5
Uknown	Yes	2009/1/12	7.5	2010/2/9	Dead	20.4
SD	Yes	2003/6/12	9.5	2003/8/11	Dead	11.5
Uknown	No		81.9	2015/1/9	Alive	81.9
PR	No		64	2015/2/18	Alive	64
PD	Yes	2010/10/18	3.2	2011/11/8	Dead	15.9
CR	Yes	2012/10/30	11.8	2015/5/11	Alive	42.1
PR	Yes	2011/6/22	8.9	2012/5/31	Dead	20.2
PR	Yes	2012/4/3	7.7	2012/10/1	Dead	13.7
SD	Yes	2013/2/1	10.8	2014/4/10	Dead	25
PR	Yes	2013/9/3	9.6	2015/4/20	Alive	29.1
PR	Yes	2010/12/2	8.8	2013/3/15	Dead	36.2
PR	Yes	2008/12/1	10.1	2009/6/1	Dead	16

PR	Yes	2015/5/13	34.6	2015/5/13	Dead	34.6
PR	Yes	2013/9/4	10.8	2014/1/15	Dead	15.2
PR	Yes	2011/5/16	11	2011/7/6	Dead	12.6
PR	Yes	2012/4/23	8.4	2012/6/1	Dead	9.7
PD	Yes	2011/11/6	1.5	2011/12/31	Dead	1.5
SD	Yes	2013/10/25	7.6	2015/4/30	Alive	25.7
PR	No		6.9	2014/1/8	Dead	6.9
PR	Yes	2012/6/5	9.8	2012/11/12	Dead	15.1
PD	Yes	2009/6/26	4.9	2009/7/21	Dead	5.7
PR	Yes	2008/9/3	8.5	2008/10/10	Dead	9.7
SD	Yes	2011/2/23	10	2013/4/16	Dead	35.7
PD	Yes	2006/7/20	2.8	2007/7/28	Dead	15
PD	Yes	2010/2/18	3.3	2011/8/30	Dead	21.6
SD	Yes	2007/12/4	3.7	2008/4/2	Dead	7.6
PD	Yes	2011/6/26	0.8	2011/6/26	Dead	0.8
PR	Yes	2011/10/20	16.9	2012/8/6	Dead	26.4
PR	Yes	2008/3/17	9	2010/7/7	Dead	36.6
SD	Yes	2012/12/28	14.2	2013/6/15	Dead	19.7
SD	Yes	2015/5/7	50.4	2015/5/7	Alive	50.4
SD	Yes	2011/2/1	9	2013/7/11	Dead	38.2
PR	Yes	2011/9/6	11.7	2014/1/31	Dead	40.5
PR	Yes	2011/6/16	10.4	2011/12/5	Dead	16.1
SD	Yes	2012/10/9	15.7	2015/5/4	Alive	46.5
SD	Yes	2009/9/21	14.9	2010/3/23	Dead	20.9
PD	Yes	2008/7/31	1.8	2009/5/12	Dead	11.2
SD	Yes	2015/1/29	23.2	2015/5/6	Alive	26.4
SD	Yes	2008/11/20	10.8	2009/12/17	Dead	23.7
PR	Yes	2011/10/19	8.5	2012/3/20	Dead	13.5
PD	Yes	2007/11/21	0.7	2007/11/22	Dead	0.8
PD	Yes	2009/10/29	2.3	2009/11/22	Dead	3.1
PR	Yes	2013/8/30	7	2015/1/27	Dead	23.9
PR	Yes	2013/11/15	12.3	2014/7/16	Dead	20.3
SD	Yes	2008/10/6	7	2009/5/27	Dead	14.7
SD	Yes	2008/8/26	2.9	2008/9/21	Dead	3.7
PR	Yes	2010/7/23	9.8	2012/6/25	Dead	32.9
SD	Yes	2010/4/6	7.1	2010/4/6	Dead	7.1
PR	Yes	2009/12/21	2.6	2009/12/21	Dead	2.6
PR	Yes	2013/7/1	8.2	2015/5/7	Alive	30.4
PR	Yes	2013/4/15	25.5	2013/8/17	Dead	29.6
PD	Yes	2011/10/17	1	2011/10/17	Dead	1
SD	Yes	2015/2/12	22.7	2015/4/1	Alive	24.3
PR	Yes	2010/3/16	8.3	2011/2/2	Dead	18.9
PR	Yes	2014/2/20	6.5	2015/4/30	Alive	20.8
PR	Yes	2010/12/29	5.8	2010/12/31	Dead	5.9
PR	Yes	2012/7/3	10.9	2012/7/14	Dead	11.3
PD	Yes	2009/3/3	0.9	2009/3/3	Dead	0.9
SD	Yes	2011/3/16	6.5	2011/7/12	Dead	10.4
SD	Yes	2014/10/6	10.3	2014/10/22	Dead	10.9
PR	Yes	2010/9/14	9.6	2014/12/13	Dead	60.6
PD	Yes	2009/1/14	0.9	2009/1/14	Dead	0.9

PR	Yes	2010/6/15	17.8	2011/11/14	Dead	34.8
PD	Yes	2012/3/29	1.6	2012/8/28	Dead	6.6
PD	Yes	2007/10/18	3	2008/8/20	Dead	13
Uknown	No	2013/7/8	32.4	2015/5/11	Alive	54.5
PR	Yes	2011/7/7	9.1	2013/5/29	Dead	31.8
PR	No		77.2	2015/3/6	Alive	77.2
PR	Yes	2009/4/10	6	2009/7/21	Dead	9.3
PR	Yes	2013/6/3	8.3	2014/10/5	Dead	24.3
PD	Yes	2009/3/24	2.8	2010/2/16	Dead	13.6
PD	Yes	2010/8/2	2.8	2010/10/12	Dead	5.1
SD	Yes	2009/5/6	4.5	2013/12/19	Dead	60
PR	Yes	2010/7/20	8.8	2011/10/21	Dead	23.8
PD	Yes	2010/11/4	2.6	2012/7/28	Dead	23.4
SD	Yes	2011/4/13	6.5	2011/12/21	Dead	14.8
PR	Yes	2011/10/6	25.9	2013/7/26	Dead	47.5
SD	Yes	2012/12/12	14	2014/11/13	Dead	37.1
SD	Yes	2011/8/17	3.3	2011/8/17	Dead	3.3
PD	Yes	2008/5/5	1.9	2008/11/6	Dead	8
PR	Yes	2010/10/4	11.6	2011/8/12	Dead	21.8
SD	Yes	2012/9/10	5.7	2013/9/15	Dead	17.9
PR	Yes	2012/5/9	10.1	2012/11/28	Dead	16.8
PD	Yes	2010/10/22	2.4	2015/3/4	Alive	54.8
SD	Yes	2008/2/29	6.5	2008/4/28	Dead	8.5
PD	Yes	2011/1/10	0.9	2011/1/10	Dead	0.9
PD	Yes	2009/3/29	0.2	2009/3/29	Dead	0.2
PD	Yes	2012/10/25	2.6	2012/12/24	Dead	4.5
PD	Yes	2012/2/12	11.7	2012/2/12	Dead	11.7
PR	Yes	2013/2/12	15.8	2014/11/12	Dead	36.8
PR	Yes	2010/3/24	11.6	2014/12/11	Alive	68.2
PR	Yes	2010/1/29	10.4	2012/10/2	Dead	42.5
SD	Yes	2009/5/20	15.9	2013/5/4	Dead	63.3
PR	Yes	2009/12/28	7.5	2010/7/10	Dead	13.9
PD	Yes	2008/7/30	4.9	2009/3/6	Dead	12.1
PR	Yes	2012/11/21	10.4	2015/5/12	Alive	40
PR	Yes	2011/9/2	9.2	2012/1/27	Dead	14
PR	Yes	2001/8/21	11.1	2001/11/4	Dead	13.5
PR	Yes	2013/1/14	7.8	2013/4/27	Dead	11.1
PR	Yes	2011/5/17	8.5	2013/2/4	Dead	29.2
PR	Yes	2013/9/10	11.1	2014/2/11	Dead	16.1
PD	Yes	2012/7/24	1.6	2012/8/26	Dead	2.7
CR	Yes	2014/11/26	33.8	2015/3/12	Alive	37.3
PD	Yes	2013/1/22	3	2014/3/15	Dead	16.7
PD	Yes	2011/11/23	2.9	2012/1/15	Dead	4.7
PR	Yes	2015/3/2	24.1	2015/5/14	Alive	26.5
SD	Yes	2014/7/31	12.3	2015/5/5	Alive	21.4
PR	Yes	2011/1/25	7.4	2012/5/1	Dead	22.6
PR	Yes	2013/2/25	14.7	2014/11/27	Dead	35.7
SD	Yes	2010/9/30	5.1	2012/3/10	Dead	22.4
PD	Yes	2010/1/23	1.8	2010/1/23	Dead	1.8
PR	Yes	2013/7/5	14.1	2014/12/8	Dead	31.2

CR	Yes	2013/3/31	3.4	2013/3/31	Dead	3.4
PR	Yes	2012/3/6	6.1	2012/11/1	Dead	14
PR	Yes	2009/10/12	6.7	2013/1/28	Dead	46.2
SD	Yes	2010/7/22	4	2012/1/19	Dead	21.9
PD	Yes	2010/7/4	3.2	2010/7/16	Dead	3.5
PD	Yes	2012/1/5	1.8	2012/5/28	Dead	6.6
PR	Yes	2014/3/9	9.6	2015/3/1	Alive	21.4
PR	Yes	2012/9/26	13.5	2015/5/6	Alive	44.8
PR	Yes	2008/4/17	3.3	2009/10/8	Dead	21
PR	Yes	2008/3/20	8.3	2008/10/15	Dead	15.2
PR	Yes	2010/3/15	8	2011/1/31	Dead	18.6
PD	Yes	2013/4/12	4.5	2013/11/1	Dead	11.2
PD	Yes	2009/4/23	1.2	2009/4/23	Dead	1.2
PD	Yes	2012/1/21	3.5	2012/3/2	Dead	4.9
PD	Yes	2010/10/14	7.7	2010/12/9	Dead	9.5
SD	Yes	2012/6/28	8.3	2015/1/7	Dead	38.6
PD	Yes	2009/8/30	1.7	2009/8/30	Dead	1.7
PR	Yes	2009/5/4	18.1	2010/12/2	Dead	37.1
PD	Yes	2008/1/29	2.1	2008/3/17	Dead	3.7
SD	Yes	2013/8/9	13.2	2015/5/11	Alive	34.2
PR	Yes	2011/8/11	8.7	2012/6/14	Dead	18.9
PR	Yes	2011/6/28	12.2	2013/3/20	Dead	33
SD	Yes	2011/8/9	15.9	2011/8/24	Dead	16.4
PD	Yes	2013/12/16	5.5	2013/12/16	Dead	5.5
SD	Yes	2012/1/27	12	2013/11/16	Dead	33.6
SD	Yes	2010/1/21	13.5	2012/5/6	Dead	41
PR	Yes	2014/6/17	8.7	2014/10/21	Dead	12.8
PR	Yes	2010/6/21	6.4	2010/7/25	Dead	7.6
PR	Yes	2009/8/18	5.7	2011/12/5	Dead	33.2
PD	Yes	2012/9/24	7.7	2012/9/24	Dead	7.7
PR	Yes	2009/9/1	9.8	2011/5/4	Dead	29.9
PR	Yes	2014/10/31	27.3	2015/5/15	Alive	33.7
CR	No		55.7	2015/5/14	Alive	55.7
PR	Yes	2014/9/11	15	2015/4/13	Dead	22
PR	Yes	2009/2/11	7.8	2010/3/24	Dead	21.1
PR	Yes	2011/11/16	9.5	2015/4/28	Alive	50.8
	Yes	2015/2/18	13.7	2015/2/18	Alive	13.7
PR	Yes	2011/1/24	6.5	2012/6/20	Dead	23.4
PR	Yes	2011/1/31	7.3	2013/6/25	Dead	36.1
PD	Yes	2010/5/28	4.9	2010/10/21	Dead	4.9
PR	Yes	2006/12/29	1	2007/12/19	Dead	12.6
PR	Yes	2014/2/14	10.7	2015/1/15	Alive	21.7
PR	Yes	2010/11/2	12	2012/6/1	Dead	30.9
SD	Yes	2012/3/9	7.7	2013/4/23	Dead	21.2
PD	Yes	2012/4/6	1.9	2012/5/10	Dead	3.1
PD	Yes	2012/2/3	0.5	2012/2/3	Dead	0.5
SD	Yes	2008/3/26	6.2	2008/8/13	Dead	10.8
PD	Yes	2010/9/14	2.7	2012/5/17	Dead	22.7
SD	Yes	2012/7/23	28.7	2015/4/16	Alive	61.5
